# Awareness and perceptions of electroconvulsive therapy among psychiatric patients: a cross-sectional survey from teaching hospitals in Karachi, Pakistan

**DOI:** 10.1186/1471-244X-7-27

**Published:** 2007-06-21

**Authors:** Mehreen Arshad, Ahmad Zafir Arham, Mansoor Arif, Maria Bano, Ayisha Bashir, Munira Bokutz, Maria Maqbool Choudhary, Haider Naqvi, Murad Moosa Khan

**Affiliations:** 1 Aga Khan University, Stadium Road, P.O. Box 3500, Karachi 74800, Pakistan; 2 Department of Psychiatry, Aga Khan University, Stadium Road, P.O. Box 3500, Karachi 74800, Pakistan

## Abstract

**Background:**

Electroconvulsive therapy (ECT) is shown to be effective in many psychiatric illnesses, but its distorted projection by the Pakistani media and its unregulated use by many physicians across the country have adversely affected its acceptability. Given this situation we aimed to assess the awareness and perceptions regarding ECT as a treatment modality among the psychiatric patients.

**Methods:**

This was a questionnaire based cross-sectional study carried out at 2 tertiary care hospitals in Karachi, Pakistan.

**Results:**

We interviewed 190 patients of which 140 were aware of ECT. The study showed that the level of education had a significant impact on the awareness of ECT (p = 0.009). The most common source of awareness was electronic and print media (38%), followed by relatives (24%) and doctors (23%). Physical injuries (42%) and neurological (12%) and cognitive disturbances (11%) were the commonly feared side effects. The most popular belief about ECT was that it was a treatment of last resort (56%). Thirty-nine percent thought that ECT could lead to severe mental and physical illness and 37% considered it inhumane. Patients' willingness to receive ECT was dependant on whether or not they were convinced of its safety (p = 0.001) and efficacy (p = 0.0001).

**Conclusion:**

We identified a serious lack of dissemination of information regarding ECT by the psychiatrists and the mental health care providers. This may be the result of an inadequate postgraduate training in Pakistan or just a lack of concern about the mentally ill patients. The media seemed to be the major source of information for our patients. We also saw the prevalence of a variety of myths regarding ECT in our society, which we feel may be responsible for the patients' adverse attitudes. Given the widespread applicability of ECT there is a dire need to dispel these misconceptions and improve its acceptability.

## Background

Electroconvulsive therapy (ECT) was introduced in its current form by Cerletti and Bini in 1938 [1]. Since then it has been subjected to controversy and antipathy by the media and general public alike. Along with an inadequate post-graduate training in psychiatry, in some developing countries like Pakistan, it is responsible for the widespread unacceptability of this treatment form among patients.

The psychiatrist to population ratio is dismally low in Pakistan. In a recent World Health Organization (WHO) information facts sheet it was estimated to be 0.2/100,000 [2]. Proper mental health care is provided by only a few hospitals. There are 2,154 beds available in the Government mental health sector. Among these, 943 beds are allocated in three large custodial centers in the country [3]. The Governmental budgetary allocation on health sector is less than 1 % of GNP; mental health allocations are negligible. It is estimated that private sector accounts for some 70 % of national expenditure on the provision of health services [4]. This demand and supply gap is exploited by the unregulated private health sector, notorious for its use of 'unmodified' ECT (ECT conducted without the use of adequate anesthesia or muscle relaxants, which can cause fractures, tongue bites, and an enormous amount of mental and physical anguish [5]). No specific research has been carried out in Pakistan, but statistics extrapolated from neighboring India, show that despite the high risk associated with unmodified ECT, a large number of psychiatrists continue its use [5]. This has led to mistrust among the general population about ECT as an effective treatment modality. Given the unusually high prevalence of depression in Pakistan, 25% to 66% for women and 10% to 49% for men in rural areas and 10% to 25% point prevalence in urban areas [6,7], this distrust is alarming and requires immediate attention as many patients may reject an essential treatment due to their misperceptions about ECT.

There is substantial scientific literature on the efficacy and indication of ECT, and many patients in western countries have expressed their satisfaction [8-12], but there are few accounts of how it is viewed by patients, especially in a developing country like Pakistan. The few studies carried out, are on the recipients on ECT. Little is known about the patients' views before they accept the treatment. We believe it is these views that determine this treatment's acceptability. Lack of local data on patient's perceptions has also hampered efforts to advocate adequate ECT service utilization.

In view of these facts we designed the study with the objective to:

1. Assess Awareness regarding ECT among psychiatric patients

2. Study their Perceptions about ECT

3. Assess the Acceptability of ECT

## Methods

### Study design and setting

This was a questionnaire based cross-sectional study, carried out over four weeks at 2 tertiary care hospitals in Karachi, Pakistan: Aga Khan University Hospital (AKUH) and Liaquat National Post-Graduate Medical Center (LNPMC). A non-probability, convenient sampling methodology was used.

AKUH is a 500 bed, private hospital, having a 15-bed psychiatric facility. The weekly turnover in the out-patients department (OPD) is 200–250 patients. LNPMC is an 800 bed, semi-private hospital with a 10-bed psychiatric unit and a weekly OPD turnover of 140–160 patients.

### Questionnaire

The questionnaire was self-developed in order to measure the various issues that were pertinent to our society. For this locally published newspapers and magazines were searched for comments on ECT and its acceptability, and detailed discussions and peer-review between investigators and facilitating faculty was carried out.

A team of seven 4^th ^year medical students carried out the data collection. A standardized Urdu version of the questionnaire was used for patients who could not understand English. Patients were recruited from the waiting room prior to their meeting with the consultant psychiatrist, and were then interviewed in a separate room to maintain confidentiality. A brief description of the questionnaire is given in the next paragraph. The questionnaire itself is available from the authors.

The socio-demographic details of age, gender, educational status and the monthly household income were recorded. Patients were then asked if they had heard about ECT. Those who responded that they had never heard about ECT were not questioned any further. Those who were aware of it were asked what had been their source of information and in their opinion, what are the indications and side-effects of ECT.

In order to evaluate the patients' perceptions and acceptability we asked about their views on the safety of ECT and if they would agree to undergo ECT if recommended by their psychiatrists. We also inquired about their confidence in ECT relative to conventional medical treatment. A review of local literature in print-media helped us in compiling a list of 7 common beliefs about ECT. The patients were then asked which beliefs they held.

A skip pattern was designed to dichotomize the above responses into those who had received ECT and those who had not. Recipients of ECT were further asked about their source of referral, the treatment method and if their consent had been taken.

### Inclusion criteria

• Psychiatric patients, age 16 years and above, either coming for consultation or admitted in the ward at AKUH and LNPMC.

### Exclusion criteria

• Patients who could not speak the national language (Urdu) or other regional dialects (immigrants without interpreter).

• Those who were grossly psychotic or acutely manic.

• Those under influence of or being treated for substance abuse.

### Ethical consideration

The study was conducted in compliance with the 'Ethical principles for medical research involving human subjects' of Helsinki Declaration. The study protocol was approved by the Ethical Review Committee (ERC) at AKUH. Verbal informed consent was taken from our subjects before the interview. Their personal information (name, residence and Medical Record number) was not recorded to maintain anonymity and confidentiality.

### Statistical Methods

The data was analyzed using the SPSS statistical package, Version 12.0.1 (Chicago, IL, USA). Descriptive statistics were employed to determine the demographic and clinical variables. Chi square test was utilized at a confidence interval of 95% to determine if the association between two variables was real.

## Results

### Socio-demographic characteristics

A total of 190 subjects were interviewed (151 from AKUH and 39 from LNPMC). There were an almost equal number of males (n = 92) and females (n = 98). Sixty-nine (36%) patients had less than 10 years of education and 121 (64%) were educated beyond that. One hundred and sixty-five (87%) patients were diagnosed to have various forms of mood disorders, 3 suffered from substance abuse, 2 each from somatoform and psychotic disorders and the rest did not have a working diagnosis. Forty-six percent (n = 87) of the patients had been on psycho-tropic medications for more than a year.

### Awareness of ECT as a treatment modality

Of all the patients 140 (74%) had heard about ECT and 50 (26%) were unaware of this treatment. The socio-demographic details of these 2 groups are given in Table 1. Females were seen to be more unaware of this treatment modality (p = 0.06).

**Table 1:** Socio-demographic Characteristics of patients of patients who were Aware vs. Unaware of ECT as a treatment modality

	Aware		Unaware	
	N	(%)	N	(%)
Age (Years)				
16–25	33	24	11	22
26–35	33	24	13	26
36–45	30	21	11	22
46–55	28	20	5	10
56–65	12	9	5	10
> 66	4	3	5	10
				
Marital status				
Single	39	28	13	26
Married	97	69	36	72
Widowed/Divorced	4	3	1	2
				
Occupation				
Unemployed	25	18	5	10
Unskilled	5	4	0	0
Skilled	7	5	2	4
Businessman	14	10	2	4
Professional	32	23	8	16
Housewife	41	29	24	48
Student	12	9	4	8
Retired	3	2	2	4
				
Monthly Income (in US$)				
< 85	16	11	5	10
85–160	22	16	8	16
160–330	22	16	8	16
330–500	20	14	4	8
> 500	34	24	10	20

The level of education also had a significant impact on the awareness of ECT. Forty-two percent (n = 30) of the patients with an education less than 10 years did not know about ECT whereas only 22% (n = 27) of patients with a higher education were unaware of it (p = 0.009).

The most popular sources of information, for patients who were aware of ECT, were electronic and print media, relatives and friends (38 %, 24 % and 10 % respectively). Only 23% of our patients identified doctors as a source.

The majority (86%, n = 120) of these patients were convinced about the effectiveness of ECT as a treatment modality. However, 86 patients (62%) thought this procedure would have serious side effects: injuries (42% of the patients), neurological impairments (12%), cognitive disturbances (11%) and pain (8%) being the most common concerns.

When patients were asked to list all the indications of ECT, 60% (n = 84) of the patients accurately specified mood disorders as a major indication. Some commented on its utility in affective psychosis (26%, n = 36) and imminent suicide (4%, n = 6). A large number of interesting responses were categorized under "others" (33%, n = 46) including 'vascular spasm', 'dullness of senses', 'brain cancers', and 'to get rid of old memories'.

### Patients' Perceptions about ECT

We found that the majority of our patients (42%, n = 59) were skeptical of the safety of ECT as a treatment modality. Twenty-eight percent (n = 39), however, considered it completely safe, while 12% (n = 17) considered it safe only when the proper procedural guidelines were followed i.e. with anesthesia and muscle relaxants.

When we asked if patients would agree to undergo ECT if advised by their psychiatrists, 59% (n = 83) said they would not, whereas the rest were ready to comply. Patients' willingness was dependant on whether or not they were convinced of its safety (p = 0.001), and efficacy (p = 0.0001).

Fourteen percent (n = 20) of our patients had found their medications to be ineffective and interestingly this group also had greater awareness of ECT (p = 0.034). We also inquired about the patients' perception of the relative efficacy of ECT versus the conventional medical treatment, 83% considered medication to be superior.

### Common Beliefs

**Figure 1:**
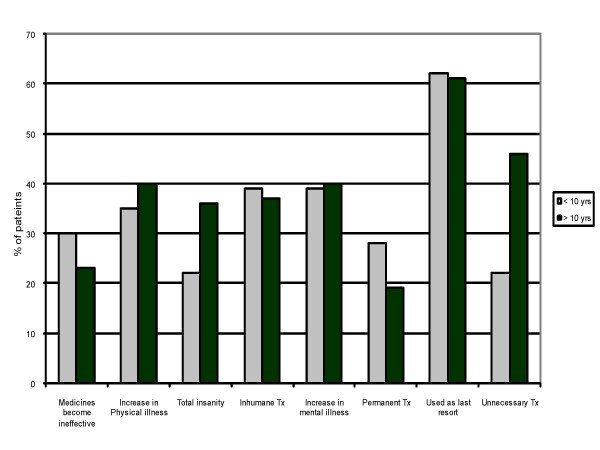
Prevalence of myths and level of education.

The most commonly held belief among our patients was that ECT is a treatment of last resort. The prevalence of the various other beliefs is shown in Table 2. Paradoxically a significantly greater proportion of patients with an education of more than 10 years thought that patients risk total insanity after ECT (p = 0.03) and that it is often given unnecessarily (p = 0.02). However, a greater proportion of patients with an education of less than 10 years considered ECT to be a permanent cure (p = 0.005). Figure 1 shows the responses for various myths when stratified according to the level of education. Gender or the source of information did not have any significant impact on the prevalence of the various beliefs.

**Table 2:** Myths regarding the Electroconvulsive therapy

Number	Patients Beliefs regarding ECT	N (%)
1	ECT is a treatment of Last resort	78 (56)
2	ECT cause severity in Mental and physical illness in the long run	55 (39)
3	ECT can cause total and irreversible insanity	48 (34)
4	ECT is a form of torture and an in-humane treatment method	52 (37)
5	ECT is used unnecessarily by Doctors for exploiting patients financially or otherwise.	48 (34)
6	ECT renders psychopharmacological treatment in- effective.	34 (24)
7	ECT is a permanent treatment for mental illnesses.	24 (17)

### Experience of ECT

Twenty-five patients had received ECT. Twenty-three of them were advised by a doctor, while 2 had taken it electively. Sixteen had been given General Anesthesia. Only 11 patients felt the procedure had been appropriately explained. Consent from the patient or their family had been taken in 20 cases. Of note patients with an education of less than 10 years were more likely to complain about deficiency in the consent process (p = 0.012).

## Discussion

We believe this is the first study in Pakistan which took into account the patients' perspective of ECT. The majority of our study population was aware of ECT and this seemed to be related to the patient's gender and education level.

### Patients' Awareness about ECT

As in previous studies, movies and media remained the most popular source of information [13,14]. And given the gruesome testimonies of former recipients of ECT that have often been published [2]; it is no wonder there is widespread fear of this treatment modality in our society.

Only a few patients had indicated psychiatrists as a source of information. Since about half the patients were on psychotropic drugs for more than a year and a substantial majority had chronic disorders, most patients had sufficient contact with their psychiatrist to discuss the option of ECT. This indicates an unsatisfactory dissemination of information by the psychiatrists. There are only 150–200 adequately trained psychiatrists in the country. Most are in private practice and located in major urban areas. Academic frauds are rampant in the government run public-sector hospitals. Officials are promoted to the highest ranks because of their political affiliations. This obviously has serious repercussions on the teaching and practices of Psychiatry in Pakistan. Inadequate training has been cited as a possible reason in some studies from other countries [14-18]. Some psychiatrists themselves have shown a negative attitude against ECT [17], which may influence their counseling about ECT.

Since there is no qualitative study from Pakistan on the benefit and efficacy of ECT, we examined this subject from the patient's perspective. Most of our patients were convinced about the efficacy of ECT, which is somewhat surprising given their main source of information. Most patients also considered it to have some side effects, especially injuries. A study on medical students in India showed similar concerns [14]. In reality, such obstacles have been overcome with the administration of anesthesia and muscle relaxants. In fact patients who have received ECT report memory impairment to be the most worrisome side effect [11,12]. Since only a few trials have studied the possibility of long-term cognitive impairment [11], there is a dire need to further investigate this aspect [22,23].

Mental health legislation in Pakistan is subject to the same complex judicial rules as so many other areas of social welfare are. Up until five years ago the judiciary was still following a modified version of the Lunacy Act of 1912. So it is no surprise that there are no national guidelines or protocols for training or clinical use of ECT, and hence no supervision of the institutes providing ECT. This has resulted in many patients either undergoing ECT unnecessarily or receiving its unmodified version with a consequent high risk of complications. This has made most patients wary of the procedure. Even though ECT has been shown to have a considerably good safety profile when procedural recommendations are followed[20,26].

### Patients' Perceptions about ECT

Malpractice of ECT in Pakistan and its inaccurate media reporting raises certain misperceptions and beliefs which play a major role in the acceptability of ECT prescription. A popular misconception is that ECT is used only as a last resort. This probably arises from the earlier false presumption, even in the developed countries, that ECT should only be used in medication-resistant patients[27]. It may also arise from the negative image of ECT in movies and media that portray it as inhumane. Studies have shown this to have adverse effects on individuals in the medical field as well[14,19]. Very few clinical audits have been carried out in order to objectively monitor the out-come of ECT prescription in Pakistan. This indiscriminate use has produced a feeling of mistrust, and many regard ECT as only a palliative treatment, with more disadvantages than benefits.

Our study showed that patients with higher education were more likely to believe that there was a possibility of total insanity after receiving ECT and that it was often used unnecessarily. This seems paradoxical at first, but it may be explained by the fact that this group has greater access to the electronic and print media, and consequently the negative image portrayed by them, which may influence their views. On the contrary, patients with lower education believed that ECT is a permanent treatment, which indicates the inadequate patient counseling, since most patients need to continue medications and subsequent relapses are not uncommon[21].

Fear of the procedure, doubts about its safety and the increased effectiveness of the newer generation of psychiatric medications has made many patients reluctant to receive ECT. Medical personnel have voiced similar opinions[17,19]. However, patients and family members from developed nations have often expressed their complete satisfaction with the procedure[8,25].

### Patients' Experiences with ECT

A number of patients had received ECT without General Anesthesia. More than half of them complained about receiving inadequate information before the procedure. Similar patient dissatisfaction has also been noted in other studies, but it was suggested that this may be due to the patient's mental state which made it difficult to understand and retain new information [12]. Like previous studies deficiencies in the consent process were seen[8]. This re-enforces the general perception that many physicians disregard their patient's opinion on treatment modalities[3,12]. However any conclusion in this regard should be taken with caution because of the few respondents in this category.

## Conclusion

Our study highlights a dire need to improve the acceptability of this important yet inappropriately utilized method of treatment for psychiatric disorders in Pakistan. There is an urgent need for more research in this area and we propose that patient survey similar to this be carried out at other centers in Pakistan too. Along with this, the media that serves as the primary information source for the common public should be employed constructively for raising public awareness and projecting ECT in a positive light. This will help in devising programs, which will dispel the commonly held myths and misconceptions about ECT, thereby improving its acceptability.

## Competing interests

The author(s) declare that they have no competing interests.

## Authors' contributions

MA conceived the study and participated in its designing, data collection and literature search. She also carried out statistical analysis and drafted the manuscript. HN participated in the study design, statistical analysis, drafting and editing the manuscript. AZA and MA participated in developing the questionnaire, data collection and statistical analysis. MB, AB and MB participated in the study design, data collection and literature search. MMC participated in the study design, data collection and helped in the drafting the manuscript. MMK participated in study design, statistical analysis and editing of the draft.

## Pre-publication history

The pre-publication history for this paper can be accessed here:


